# Detection of spheno-occipital synchondrosis fusion stages using artificial intelligence

**DOI:** 10.3389/fphys.2025.1682917

**Published:** 2025-11-12

**Authors:** Sultan Uzun, Guldane Magat, Cengiz Evli

**Affiliations:** 1 Department of Dentomaxillofacial Radiology, Necmettin Erbakan University Faculty of Dentistry, Konya, Türkiye; 2 Department of Dentomaxillofacial Radiology, Ankara University Faculty of Dentistry, Ankara, Türkiye

**Keywords:** growth and development, craniofacial anomaly, spheno-occipital synchondrosis, YOLO, deep learning, artificial intelligence

## Abstract

**Introduction:**

Accurate evaluation of the spheno-occipital synchondrosis (SOS) is important for growth assessment, early detection of craniofacial anomalies, and reliable forensic age estimation.

**Methods:**

This study applied three deep learning models—YOLOv5, YOLOv8, and YOLOv11—to cone-beam computed tomography (CBCT) sagittal images from 1,661 individuals aged 6–25 years, aiming to automate SOS fusion stage classification. Model performance was compared in terms of detection accuracy and processing speed.

**Results:**

All models achieved high accuracy, with a mean average precision (mAP) of 0.995 in complete fusion (Stage 3). YOLOv8 demonstrated the most consistent balance of precision and recall, while YOLOv11 achieved the fastest inference time (27 ms). YOLOv5 excelled in specific stages with perfect F1-scores.

**Discussion:**

These findings demonstrate that YOLO-based AI models can provide precise, rapid, and reproducible SOS assessments, offering valuable support for both clinical decision-making and forensic investigations.

## 1 Introduction

Craniofacial growth is a complex biological process influenced by genetic, environmental, and functional factors. The three synchondroses of the cranial base—spheno-ethmoidal, inter-sphenoid, and spheno-occipital synchondroses (SOS)are significant growth centers, with the SOS being the last to close (Krishan and Kanchan, 2013; Singh et al., 2025). The spheno-occipital synchondrosis (SOS), a cartilaginous joint between the sphenoid and occipital bones, plays a crucial role in this process. As a key growth center, SOS facilitates the extension of the cranial base axis, enabling the development of teeth and alveoli and thus contributing to craniofacial formation (Dalili Kajan et al., 2021; Funato et al., 2020).

The cranial base represents the first stable structure in craniofacial growth, rather than the occipital bone. The maxilla usually grows downward and forward as described by classic implant studies (Björk, 1955; Melsen, 1974; Solow, 1980), and its positional changes are largely influenced by remodeling and displacement relative to the cranial base rather than direct growth at the spheno-occipital synchondrosis (SOS). Growth at the SOS primarily contributes to the elongation of the posterior cranial base and the flexure angle between the anterior and posterior cranial base, which in turn may affect the spatial relationship of the mandible and temporal bone relative to the cranial base (Halpern, 2014; Demirturk Kocasarac et al., 2016; Sinanoglu et al., 2016; Cendekiawan et al., 2010; Nie, 2005).

In skeletal Class III malocclusion, early fusion or reduced growth potential at the SOS has been linked to a shorter posterior cranial base and a more acute cranial base angle, leading to a retruded maxilla and a forward-positioned mandible (Singh et al., 2025; Halpern, 2014; Cendekiawan et al., 2010). Clinical and imaging studies have consistently demonstrated distinctive cranial base features in Class III patients, such as shorter posterior cranial base length during the prepubertal period and altered mandibular positioning (Yang et al., 2016; Singh et al., 2025). However, Yang et al. reported that the timing and fusion patterns of the SOS were not significantly different between Class I and Class III groups, suggesting that while SOS maturation contributes to cranial base morphology, it is unlikely to act as the sole determinant of sagittal jaw discrepancies.

Thus, the importance of SOS fusion and growth direction in Class III malocclusion lies in their potential to influence cranial base angulation and mandibular displacement, rather than directly altering maxillary growth direction. This perspective highlights the cranial base as a key mediator of skeletal pattern, whereas the maxilla continues its downward and forward growth trajectory relative to this stable base.

Fusion of the SOS occurs later than other midline synchondroses. While the spheno-ethmoidal synchondrosis (SES) typically fuses by age six and the intersphenoid synchondrosis (ISS) at birth, SOS generally fuses between ages 12 and 15 (Evli et al., 2025). However, its exact timing is debated, with reports ranging from 8 to 21 years (Nie, 2005; Hoshino et al., 2022). Because SOS is the last cranial base synchondrosis to close, it provides a unique window for growth assessment. Its maturation stages have been linked to skeletal maturity and can offer valuable insight for treatment timing, particularly in orthodontic and orthopedic interventions such as headgear therapy and rapid maxillary expansion (Halpern, 2014). If performed before SOS fusion, these techniques can temporarily open or influence the synchondrosis, facilitating maxillary displacement and alveolar adaptation (Halpern, 2014).

In craniofacial syndromes, midface hypoplasia is believed to stem from early or atypical SOS ossification and disrupted cranial base growth (Sinanoglu et al., 2016). Aberrant fusion may indicate underlying defects; in syndromes like Apert, Crouzon, and Muenke, premature SOS closure correlates with midfacial underdevelopment (Goldstein et al., 2014; McGrath et al., 2012; Tahiri et al., 2014).

Advances in artificial intelligence (AI) have enabled the use of machine learning in craniofacial research to predict growth patterns. AI aids in diagnosing craniofacial anomalies and evaluating interventions like rapid maxillary expansion (Bazargani et al., 2013; Geisler et al., 2021). Leveraging large datasets and imaging, AI improves diagnostic accuracy and treatment planning in orthodontics and craniofacial surgery.

Given the clinical importance of SOS fusion, accurately identifying its stages is key for tracking development and detecting anomalies early. Cone-beam computed tomography (CBCT) offers high-resolution imaging to assess SOS fusion precisely. This study aims to apply AI algorithms to classify SOS fusion on CBCT images (CBCTIs) and examine its correlation with growth periods. We hypothesize that AI-based evaluation will support early anomaly detection, leading to better diagnosis and treatment planning.

## 2 Materials and methods

### 2.1 Ethics approval and sample size determination

This thesis study received ethical approval from the Local Non-Drug and Non-Medical Device Research Ethics Committee on 25 January 2024, with decision number 2024/363. All procedures adhered to the principles of the Declaration of Helsinki.

Based on a one-sided independent sample t-test with a 95% confidence level, 95% test power, and an effect size of d = 0.518, it was determined that a minimum of 85 participants per group was needed (Geng et al., 2024).

### 2.2 Image collection and inclusion criteria

This retrospective study evaluated CBCT images (CBCTIs) acquired between 2020 and 2024 from the Departments of Dentomaxillofacial Radiology at Necmettin Erbakan University Faculty of Dentistry and Ankara University Faculty of Dentistry. Included were sagittal section CBCTIs from individual aged 6–25 that clearly showed the spheno-occipital synchondrosis (SOS) with high diagnostic quality. Images were excluded if they:

- Were from individuals over age 25,
- Showed congenital/acquired maxillofacial deformities,
- Had large pathological lesions or trauma history,
- Showed evidence of head and neck surgery, radiotherapy, or chemotherapy,
- Came from syndromic cases impacting the craniofacial region,
- Or had insufficient resolution, artifacts, or incomplete SOS depiction.

To ensure image quality and consistency, all CBCTIs were standardized. Technical settings and imaging protocols were selected to minimize variables that could affect SOS visibility.

### 2.3 Radiographic processing, data labeling and preparation

Images were acquired using three CBCT devices: J. Morita 3D Accuitomo 170, Newtom Go, and Newtom Giano HR, all operating at 90 kVp, 5 mA, 17.5 s, with a 0.25 mm voxel size. DICOM files (.dcm) were viewed on a 27-inch UltraSharp LED TFT screen (2560 × 1440, 3.7 MP). Sagittal slices showing SOS were saved as 2D JPEG images (600 dpi, 1024 × 640 pixels) after contrast and brightness adjustments for optimal AI input standardization.

From 262 CBCT datasets, 1,661 sagittal 2D cross-sectional images were extracted. These were classified into four SOS fusion stages based on the Fernández-Pérez et al. (2016) system:

- Stage 0: No fusion,
- Stage 1: Endocranial fusion visible,
- Stage 2: Ectocranial fusion observed,
- Stage 3: Complete fusion with no gap.

The distribution was as follows: 379 images (Stage 0), 725 (Stage 1), 144 (Stage 2), and 413 (Stage 3).

In our study, all dataset labeling and model development were performed through the CranioCatch artificial intelligence platform (accessible at https://dentalai.ogu.edu.tr/), which is a web-based system designed for medical and dental imaging analysis. This platform provides tools for image annotation, dataset management, and AI model training, eliminating the need for direct coding by the researchers. All data were anonymized before being uploaded to the CranioCatch platform (Eskişehir, Türkiye).

**FIGURE 1:**
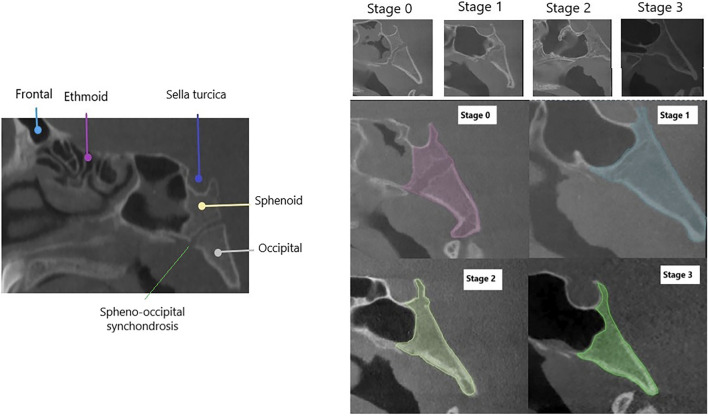
SOS fusion stages and polygonal segmentations of stages.

Images were labeled using polygonal segmentation in CranioCatch. Structures like the sphenoid body, SOS, and occipital bone were outlined, including cortical boundaries. Labeling was done in four classes (Stage 0–3) (Figure 1). All segmentations were reviewed by two observers—one with 7 years and another with 15 years of experience. Intra- and inter-observer agreement values were excellent (0.995 and 0.983, respectively) (Table 1).

**TABLE 1:** Performance metrics calculated using 50% IoU threshold of YOLOv5, YOLOv8, and YOLOv11 models, with comparable values from experts and AI models.

AI models							Experts						
**YOLOv5**	**TP**	**FP**	**FN**	**Sensitivity**	**Accuracy**	**F1 Score**	**Intra-observer**	**TP**	**FP**	**FN**	**Sensitivity**	**Accuracy**	**F1 Score**
General	154	12	0	1	0.92771084	0.9625							
Stage 0	27	6	6	0.81818182	0.81818182	0.81818182							
Stage 1	68	5	5	0.93150685	0.93150685	0.93150685							
Stage 2	13	1	1	0.92857143	0.92857143	0.92857143	General	1,652	4	5	0.994	0.995	0.995
Stage 3	46	0	0	1	1	1	Stage 0	300	0	0	1.000	1.000	1.000
**YOLOv8**	**TP**	**FP**	**FN**	**Sensitivity**	**Accuracy**	**F1 Score**	Stage 1	580	2	3	0.991	0.991	0.991
General	153	13	13	0.92168675	0.92168675	0.9216867	Stage 2	118	1	0	0.992	0.992	0.992
Stage 0	29	4	4	0.87878788	0.87878788	0.8787878	Stage 3	325	0	0	1.000	1.000	1.000
Stage 1	68	5	5	0.93150685	0.93150685	0.9315068	**Inter-observer**	**TP**	**FP**	**FN**	**Sensitivity**	**Accuracy**	**F1 Score**
Stage 2	13	1	1	0.92857143	0.92857143	0.9285714							
Stage 3	43	3	3	0.93478261	0.93478261	0.9347826							
**YOLOv11**	**TP**	**FP**	**FN**	**Sensitivity**	**Accuracy**	**F1 Score**							
General	157	9	9	0.94578313	0.94578313	0.9457831	General	1,633	13	15	0.983	0.983	0.983
Stage 0	31	2	2	0.93939394	0.93939394	0.9393939	Stage 0	300	0	0	1.000	1.000	1.000
Stage 1	69	4	4	0.94520548	0.94520548	0.9452055	Stage 1	567	9	9	0.969	0.969	0.969
Stage 2	12	2	2	0.85714286	0.85714286	0.8571429	Stage 2	115	3	1	0.966	0.966	0.966
Stage 3	45	1	1	0.97826087	0.97826087	0.9782609	Stage 3	325	0	0	1.000	1.000	1.000

IoU, intersection over union; TP, true positive; FP, false positive; FN, false negative.

Preprocessing included sharpening unclear images and resizing for model training. Finalized data were split into training (1,329), validation (166), and testing (166) subsets. Specific allocations were:

- Stage 0: 300 train, 46 validation, 33 test
- Stage 1: 585 train, 67 validation, 73 test
- Stage 2: 119 train, 11 validation, 14 test
- Stage 3: 325 train, 42 validation, 46 test

### 2.4 Segmentation model training

Images were resized to 1024 × 640 pixels for training with convolutional neural network (CNN) models on the PyTorch platform. YOLOv5, YOLOv8, and YOLOv11—modern, single-stage object detection algorithms—were used due to their speed and accuracy.

Each model underwent 600 training steps, using Stochastic Gradient Descent (SGD) with a batch size of 4. The most successful training step was saved as “best.pt” (124.9 MB). During the test phase, IoU and stability threshold values were set to 0.5.

### 2.5 Model performance evaluation

Model success was assessed with a confusion matrix, comparing AI predictions to expert-labeled data. Key evaluation metrics included:

- True Positive (TP): Correct identification of fusion stages.
- False Positive (FP): Incorrect classification of non-fusion regions.
- False Negative (FN): Missed detections of actual fusion areas.

From these, the following performance metrics were calculated:

- Sensitivity (Recall) = TP/(TP + FN): Indicates the model’s ability to correctly detect SOS fusion.
- Precision = TP/(TP + FP): Reflects how many identified regions were truly SOS fusion stages.
- F1 Score = 2 × (Precision × Recall)/(Precision + Recall): Balances precision and recall for overall accuracy.
- Mean Average Precision (mAP): A benchmark metric that summarizes model performance across multiple thresholds and is widely used in object detection tasks.

These metrics collectively ensured a thorough validation of model reliability and diagnostic utility.

## 3 Results

### 3.1 YOLOv5 labeling model training and test results

In the YOLOv5 model, training was conducted using 1,329 images, and performance was tested on 166 images. Key training metrics showed that the train/box_loss and val/box_loss values were 0.01084 and 0.00726, respectively, indicating high segmentation accuracy. Similarly, train/cls_loss and val/cls_loss were 0.00735 and 0.00436, suggesting effective object classification. The gradual decrease in loss values across epochs reflected a successful learning process.

**FIGURE 2:**
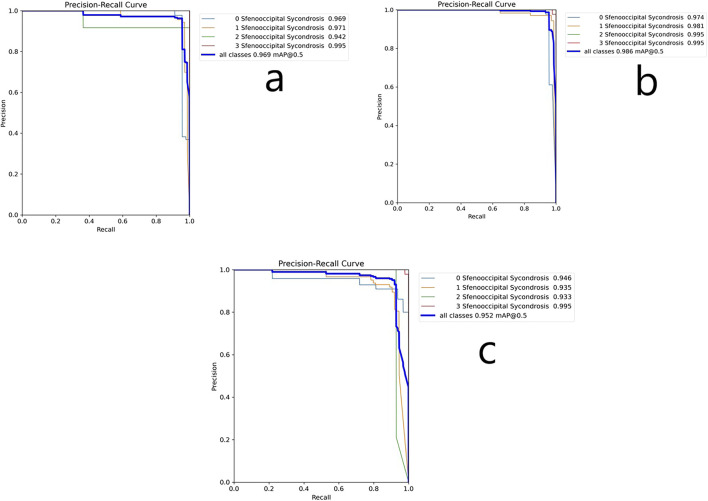
**(a)** Precision-sensitivity curve of the YOLOv5. **(b)** Precision-sensitivity curve of the YOLOv8. **(c)** Precision-sensitivity curve of the YOLOv11.

Figure 2 presents the precision-sensitivity curve at the 0.5 IoU threshold. The largest area under the curve was observed in Stage 3, followed by Stage 1, Stage 0, and Stage 2. The average mAP value was 0.969, showing strong model performance. High precision reflects a low FP rate, and high sensitivity indicates a low FN rate.

Model outputs were evaluated as True Positive (TP), False Positive (FP), or False Negative (FN) depending on their correspondence with expert annotations (Table 1). Confusion matrix analysis revealed an overall F1-score of 0.9625. The highest performance was observed in Stage 3, with 100% recognition. Stage 1 and Stage 2 followed, while Stage 0 had the lowest performance (F1 = 0.81).

### 3.2 YOLOv8 labeling model training and test results

YOLOv8 was also trained with 1329 images and tested on 166. Training metrics showed train/box_loss = 0.31148 and val/box_loss = 0.46831, with train/cls_loss = 0.31394 and val/cls_loss = 0.29065. These values, though higher than YOLOv5’s, remained low and indicated stable training. A unique metric in YOLOv8, dfl_loss, was 0.86602 (train) and 1.0124 (val), suggesting the model effectively adapted to detecting features at varying shapes and sizes.

Figure 2 shows that YOLOv8 reached its highest mAP (0.995) in Stages 2 and 3, followed by Stage 1 (0.981) and Stage 0 (0.974). The average mAP across all stages was 0.986, indicating excellent detection ability.

Evaluation metrics classified model predictions as TP, FP, or FN, compared to radiologist labels (Table 1). The overall F1-score was 0.92. Stage 3 achieved the highest F1 (0.9347), followed closely by Stage 1 (0.9315), Stage 2 (0.92), and Stage 0 (0.87), indicating consistently high performance across all fusion stages.

### 3.3 YOLOv11 labeling model training and test results

The YOLOv11 model was trained and validated using the same data distribution. During training, the train/box_loss and val/box_loss values were 0.36598 and 0.47306, respectively, and train/cls_loss and val/cls_loss were 0.33717 and 0.35153. The dfl_loss values were 0.88558 (train) and 0.99697 (val), reflecting high adaptability with minimal error in handling complex image structures.

As shown in Figure 2, YOLOv11 achieved the highest mAP in Stage 3 (0.995), followed by Stage 0 (0.946), Stage 1 (0.935), and Stage 2 (0.933). The overall average mAP was 0.952.

According to model evaluation (Table 1), YOLOv11 achieved an overall F1-score of 0.94. The stage-wise breakdown revealed Stage 3 had the highest F1 (0.97), followed by Stage 1 (0.94), Stage 0 (0.93), and Stage 2 (0.85). Although its accuracy was slightly lower than YOLOv8, it maintained strong consistency across most categories.

### 3.4 Comparison of performance times of YOLO models

Table 2 summarizes the processing times of the three models. YOLOv5 recorded the longest average time at 40 ms per image, followed by YOLOv8 at 30 ms, and YOLOv11 with the shortest time of 27 ms. Although YOLOv11 offered faster inference, YOLOv8 showed better overall balance between speed and accuracy.

**TABLE 2:** Comparison of the results of YOLO models according to SOS fusion stages.

Stages	YOLOv5	YOLOv8	YOLOv11
Stage 0	mAP: 0.969	mAP: 0.974	mAP: 0.946
	F1: 0.81	F1: 0.878	F1: 0.939
Stage 1	mAP: 0.971	mAP: 0.981	mAP: 0.935
	F1: 0.93	F1: 0.931	F1: 0.945
Stage 2	mAP: 0.942	mAP: 0.995	mAP: 0.933
	F1: 0.92	F1: 0.928	F1: 0.85
Stage 3	mAP: 0.995	mAP: 0.995	mAP: 0.995
	F1: 1	F1: 0.934	F1: 0.9782

### 3.5 Comparison of YOLO models by SOS fusion stage

As detailed in Table 2, YOLOv8 consistently outperformed the others in accuracy and stability across SOS fusion stages. For Stage 0, YOLOv8 had the highest mAP (0.974), followed by YOLOv5 (0.969) and YOLOv11 (0.946). In Stage 1, YOLOv8 again led with a mAP of 0.981. For Stage 2, YOLOv8 achieved a peak mAP of 0.995, outperforming both YOLOv5 and YOLOv11. In Stage 3, all models reached a shared maximum mAP of 0.995, though YOLOv5 achieved a perfect F1 score of 1, showing its strength in this stage.

## 4 Discussion

Since the beginning of civilization, technological progress has significantly eased workloads. Innovations like electronics, automobiles, computers, and the Internet have transformed numerous sectors, including healthcare, education, and media. Dentistry, which increasingly utilizes digital workflows, has also embraced artificial intelligence (AI) to improve diagnosis, treatment planning, image interpretation, patient management, and automation, thus enhancing oral healthcare quality (Kaya and Koc, 2024; Rahim et al., 2024).

Accurate diagnosis and planning are crucial in clinical decision-making. Studies show that AI-assisted cephalometric analysis (CA) offers more consistent results than manual methods (Lin et al., 2021; Nishimoto et al., 2020; Rahim et al., 2024). In patients undergoing skeletal maturation, AI also helps estimate growth rate and development by analyzing skeletal age, cervical vertebrae, skeletal class, and surgical outcomes (Amasya et al., 2020; Lin et al., 2021; Yu et al., 2020). These capabilities are also valuable in forensic dentistry, especially for age estimation (De Tobel et al., 2017).

Dental age and vertebral development are key in orthodontic planning, particularly since the spheno-occipital synchondrosis (SOS) is the last cranial synchondrosis to fuse. Conventional skeletal maturation indicators such as the hand-wrist (HW) and cervical vertebrae maturation (CVM) methods have been widely applied, but both present significant limitations. The HW method requires expert knowledge, is time-consuming, has moderate accuracy, and exposes patients to additional radiation. The CVM method, while more convenient, suffers from poor reproducibility, heavy reliance on clinician experience, and limited ability to predict craniofacial growth, especially in female patients with Class II malocclusion. Consequently, neither method guarantees a fully reliable tool for skeletal age assessment, and the orthodontic community recognizes the need for more accurate alternatives (Al-Gumaei et al., 2023).

Clinically, the accurate evaluation of craniofacial growth and treatment response requires stable reference structures for superimposition. Traditional cephalometric superimposition techniques rely on landmarks such as sella, nasion, or basion, but these are subject to growth-related positional changes, which reduces precision and introduces systematic error. Recent advances such as Digital Image Correlation (DIC) applied to cephalometric imaging enable superimposition on growth-stable cranial base structures without reliance on landmarks. DIC with Walker’s Point Line Combination (WPLC) has shown the highest precision, surpassing manual and conventional methods. This suggests that AI-driven approaches based on cranial base maturation can reduce observer bias, improve reproducibility, and allow more accurate longitudinal monitoring of growth and treatment outcomes. Looking ahead, combining AI-based SOS classification with advanced digital superimposition methods like DIC may create a comprehensive growth analysis framework that integrates the strengths of CBCT-based SOS staging with stable cranial base references, ultimately providing a reproducible tool for orthodontic and surgical applications (Danz et al., 2024).

In this context, SOS evaluation with CBCT represents a promising approach, as it provides high-resolution three-dimensional imaging of cranial base maturation and offers a valid and reliable indicator of skeletal maturity compared with HW, CVM, and chronological age (Al-Gumaei et al., 2023). Beyond its diagnostic accuracy, integrating AI models to classify SOS fusion stages on CBCT images may enhance orthodontic assessment, improve the prediction of craniofacial syndromes, and support more precise evaluation of developmental completion. Moreover, this approach holds potential value in forensic applications, where accurate skeletal maturity assessment is essential.

The SOS is a cartilaginous joint between the sphenoid and occipital bones and serves as a critical cranial base growth center (Alhazmi et al., 2017). Its timely fusion shapes cranial base morphology and impacts midfacial development. Premature fusion has been linked to midface hypoplasia (Tahiri et al., 2014), and its timing is crucial for adolescent age estimation in forensic science (Sinanoglu et al., 2016). However, studies vary in SOS fusion timelines due to differing methodologies like autopsy, histology, and imaging, including CT and CBCT (Kahana et al., 2003). Among these, 3D imaging modalities, especially CBCT—offer greater accuracy due to high-resolution capabilities (Alhazmi et al., 2017).

Despite the importance of SOS fusion assessment, no standard staging system is universally accepted. Different studies use varied classifications (Bassed et al., 2010; Franklin and Flavel, 2014; Shirley and Jantz, 2011). Our study adopts Franklin and Flavel (2014) four-stage classification system. Fusion generally occurs between ages 11–14 in females and 13–16 in males (Alhazmi et al., 2017), yet collecting sufficient data remains difficult due to CBCT’s limited use in children because of radiation exposure risks.

Comparable findings were reported by Al-Gumaei et al. (2023), who also applied the Franklin and Flavel classification system but labeled the stages from 1 to 4 instead of 0–3. When aligning their Stage 1 with our Stage 0, their results demonstrated that SOS maturation stages represent valid and reliable indicators of maxillary skeletal growth in both genders. Notably, they observed greater increases in maxillary length and height between stages 2 and 3 than between earlier or later stages, whereas changes in maxillary width were more pronounced between stages 1 (our Stage 0) and 2. Growth activity appeared to peak while the SOS was still fusing (particularly stages 2 and 3), with reduced increments after complete fusion (stage 4). Moreover, female patients exhibited earlier acceleration of growth compared with males when assessed by chronological age, although this sex difference was not observed when staging was based directly on SOS maturation. These findings reinforce the clinical relevance of SOS staging as a practical indicator of skeletal maturity, highlighting its potential to optimize treatment timing in orthodontic and orthopedic interventions. In addition, Geng et al. (2024), using the Lottering SOS classification, provided further insight into maxillomandibular growth dynamics across fusion stages. They found that in girls, sagittal maxillary growth remained active until SOS stage 3, slowed at stages 4–5, and continued to decline at stages 5–6. In boys, sagittal maxillary growth was stable until stage 4, with deceleration beginning from stages 5–6. Mandibular growth in both genders followed a pattern of increasing, accelerating, and then decelerating relative growth rates (RGRs) across SOS stages 2–6, with peak mandibular length observed between stages 3–4 and 4–5. These results highlight that SOS maturation reflects not only maxillary but also mandibular growth potential, further underscoring its clinical significance in timing interventions.

As object detection technologies have evolved, convolutional neural networks (CNNs) have replaced earlier algorithms. CNNs offer higher accuracy, particularly with large datasets and adequate computing power (Zhang and Hong, 2019). Object detection models are grouped into single-stage (e.g., YOLO, SSD) and two-stage (e.g., RCNN, Faster RCNN) approaches. Single-stage models prioritize speed with acceptable accuracy, while two-stage models are more precise but slower (Jegham et al., 2024; Vijayakumar and Vairavasundaram, 2024).

This study utilized three Ultralytics-supported single-stage models—YOLOv5, YOLOv8, and YOLOv11. The original YOLO (You Only Look Once) introduced by Redmon (2016) revolutionized object detection by predicting bounding boxes and class probabilities simultaneously (Hussain, 2023). To maintain comparability, unsupported versions (e.g., YOLOv1, v2, v4, v6, v7) were excluded due to architectural differences (Jegham et al., 2024).

YOLOv5, launched in 2020 by Glen Jocher, introduced CSPDarknet as a backbone, improving computational efficiency (Ultralytics, 2021; Jocher et al., 2020). YOLOv8 (2023) added the C2f module and advanced context fusion for enhanced object detection (Jegham et al., 2024). YOLOv11 (2024) further incorporated the C2PSA module—combining partial structures and self-attention for better detection of small or obscured features (Jocher and Qiu, 2024).

Mean average precision (mAP) is the preferred evaluation metric in object detection due to class imbalance challenges (Vijayakumar and Vairavasundaram, 2024). In our results, YOLOv5 yielded mAP 0.969 and F1-score 0.9625; YOLOv8 achieved mAP 0.986 and F1-score 0.9216; YOLOv11 reached mAP 0.952 with F1-score 0.945. YOLOv8 performed most consistently and accurately, aligning with previous findings (Fitria et al., 2024; Deepho et al., 2024; Bonfanti-Gris et al., 2024).

Though YOLOv11 had the fastest inference time (27 ms), it showed greater accuracy and variability, raising stability concerns. Özcan et al. (2024) similarly observed YOLOv8 outperforming YOLOv11 in dental landmark detection. Despite YOLOv11’s efficient C3k2 architecture, YOLOv8 maintained superior reliability.

All models showed peak performance in Stage 3 detection (mAP: 0.995), likely due to the distinct radiographic signs of complete fusion. While results varied in other stages, YOLOv8 outperformed others, and YOLOv11 had the lowest sensitivity.

In this study, experts achieved slightly higher sensitivity and accuracy than the AI models, particularly in Stage 0 and Stage 3, where their performance was perfect. These differences are expected, as the AI models were trained on expert-labeled data, thereby validating the reliability of the ground truth used for training. Most FN and FP results produced by the AI corresponded to borderline cases or image artifacts, which are typically recognizable by experienced observers. This suggests that AI errors are not arbitrary but remain visually interpretable, supporting the complementary role of expert review. For this reason, the most effective diagnostic workflow would involve AI providing a preliminary classification subsequently reviewed and confirmed by experts, combining the reproducibility and efficiency of AI with the diagnostic assurance of human expertise. It should also be noted that AI performance was calculated on the test dataset, whereas expert sensitivity and specificity were derived from the entire dataset, limiting direct comparability. Slightly higher values in expert evaluation should therefore be seen not as a shortcoming of AI but as confirmation of the reliability of expert annotations. The high concordance between experts and AI highlights the reproducibility of the system and its potential to replicate expert-level staging in a rapid and automated manner.

The main study limitation was the difficulty of assembling a large, balanced dataset due to age restrictions and radiation concerns. Pediatric images also showed motion artifacts and anatomical variation, affecting generalizability. Still, the models performed robustly. Future work should involve larger, multi-center datasets to validate these findings.

## 5 Conclusion

Ultralytics’ YOLO models (YOLOv5, YOLOv8, and YOLOv11) accurately detect SOS fusion stages in CBCT images, with mAP scores above 95% and F1-scores over 90%. These AI-based approaches enhance growth monitoring and early diagnosis of craniofacial anomalies. YOLOv8’s superior performance highlights the importance of model selection in improving treatment outcomes. The study demonstrates the potential of deep learning in medical imaging and suggests that future research with larger datasets and broader clinical applications could lead to widespread clinical adoption of these models.

## Data Availability

The datasets generated and analyzed during the current study are not publicly available due to privacy and ethical restrictions but are available from the corresponding author on reasonable request.
